# Protein tyrosine phosphatase 1B in metabolic and cardiovascular diseases: from mechanisms to therapeutics

**DOI:** 10.3389/fcvm.2024.1445739

**Published:** 2024-08-22

**Authors:** Yan Sun, Frank A. Dinenno, Peiyang Tang, Maria I. Kontaridis

**Affiliations:** ^1^ Department of Biomedical Research and Translational Medicine, Masonic Medical Research Institute, Utica, NY, United States; ^2^ Department of Medicine, Division of Cardiology, Beth Israel Deaconess Medical Center, Boston, MA, United States; ^3^ Department of Biological Chemistry and Molecular Pharmacology, Harvard Medical School, Boston, MA, United States

**Keywords:** protein tyrosine phosphatase 1B (PTP1B), cardiovascular disease, insulin resistance, diabetes, obesity, therapeutics

## Abstract

Protein Tyrosine Phosphatase 1B (PTP1B) has emerged as a significant regulator of metabolic and cardiovascular disease. It is a non-transmembrane protein tyrosine phosphatase that negatively regulates multiple signaling pathways integral to the regulation of growth, survival, and differentiation of cells, including leptin and insulin signaling, which are critical for development of obesity, insulin resistance, type 2 diabetes, and cardiovascular disease. Given PTP1B's central role in glucose homeostasis, energy balance, and vascular function, targeted inhibition of PTP1B represents a promising strategy for treating these diseases. However, challenges, such as off-target effects, necessitate a focus on tissue-specific approaches, to maximize therapeutic benefits while minimizing adverse outcomes. In this review, we discuss molecular mechanisms by which PTP1B influences metabolic and cardiovascular functions, summarize the latest research on tissue-specific roles of PTP1B, and discuss the potential for PTP1B inhibitors as future therapeutic agents.

## Introduction

Obesity has reached epidemic proportions worldwide, with over 70% of the US population classified as overweight, obese, or morbidly obese. Projections from the World Health Organization indicate that by 2030, half of the US population will be considered obese, with children being the most vulnerable demographic (1). Obesity significantly increases the risk of developing several diseases, including certain cancers, type 2 diabetes (T2D), and cardiovascular disease (CVD) (2). Obesity adversely affects glucose and lipid levels, raises arterial blood pressure, induces inflammation, and impairs pulmonary function, which can lead to cardiac hypertrophy, atrial fibrillation, and heart failure if untreated (3–6). Despite understanding the contributing factors, the molecular mechanisms linking obesity to CVD remain poorly understood.

Primarily driven by high-calorie diets, sedentary lifestyles, and stress, insulin resistance is linked to the development of T2D, metabolic syndrome and chronic low-grade inflammation (7, 8). Indeed, the global prevalence of T2D, which accounts for 90%–95% of all diabetic cases, has surged by approximately 90% from 1990 to 2021 and is expected to increase an additional 60% by 2050 to affect more than 1.31 billion people (9, 10). This rapid rise in T2D, characterized by the dysregulation of glucose homeostasis, poses significant challenges for global health systems.

Insulin resistance is also a primary risk factor for CVD, non-alcoholic fatty liver disease (NAFLD), and hypertension (11, 12). CVD accounted for 18 million deaths in 2019 and is projected to exceed 22 million by the year 2030 (13). Atherosclerosis, characterized by lipid plaque accumulation in arterial walls, underlies most of these cardiovascular events, causing myocardial infarctions (MIs) and strokes (14). Chronic low-grade inflammation also plays a crucial role in the pathogenesis of CVD and can exacerbate systemic insulin resistance (15).

The interconnection between obesity, insulin resistance, and CVD underscores the need for comprehensive prevention and management strategies, including lifestyle changes and targeted interventions to enhance metabolic and cardiovascular health. In this regard, enzymes that modulate protein signaling pathways are crucial to maintaining cellular homeostasis; their controlled function is instrumental to preventing the development of insulin resistance, T2D, and CVD.

Protein Tyrosine Phosphatase 1B (PTP1B), encoded by the *PTPN1* gene, is a non-receptor protein tyrosine phosphatase (PTP) that has been extensively studied due to its regulatory function in insulin and leptin signaling (16, 17). As such, PTP1B plays a critical role in metabolic regulation, making it a potential target for therapeutic intervention in obesity and diabetes, as well as in CVD pathophysiology. Here, in this review, we will discuss the significance of the biochemical, functional, and mechanistic roles for PTP1B, and how its hyperactivation can lead to the development of metabolic dysfunction and CVD. We will also discuss the current and emerging therapeutic strategies for treating obesity and diabetes, including use of PTP1B inhibitors.

## PTP1B structure and function

PTP1B is a 50kDA protein initially discovered by biochemical purification methods targeting enzymes involved in protein tyrosine dephosphorylation (16, 18, 19). The C-terminus of the enzyme contains two proline-rich domains that interact with Src-homology 3 (SH3) domain-containing proteins to recruit binding substrates (19), as well as a hydrophobic domain that anchors to the endoplasmic reticulum (ER) to allow access to intracellular targets (20, 21). The N-terminal domain of PTP1B houses the catalytic domain, which includes the essential cysteine (Cys215) and arginine (Arg221) residues of the phosphatase signature motif [I/V]HCXXGXXR[S/T], a sequence that defines all protein tyrosine phosphatases (17).

The crystal structure of PTP1B has also revealed its biochemical function; it is a PTP that forms a catalytic pocket with high specificity for phosphotyrosine-containing substrates (22). Upon substrate binding, PTP1B undergoes a conformation change, closing its WPD loop around the phosphotyrosine residue to stabilize and ready the invariant aspartic acid (Asp181) for hydrolytic catalysis, a process that proceeds in two steps (23). First, a nucleophilic attack by the cysteine residue forms a phosphocysteine intermediate, which is hydrolyzed by glutamine (Gln262) and Asp181 (23). Next, the closed WPD loop sequesters the phosphocysteine intermediate to prevent the transfer of phosphate to extraneous acceptors. This process is critical to PTP1B's function, making it an integral regulator of signaling pathways involved in metabolic regulation.

## PTP1B and RTK signaling

As a PTP, PTP1B catalyzes the dephosphorylation of receptor tyrosine kinases (RTKs) to modulate multiple spatiotemporally complex signaling processes, including those mediated by epidermal growth factor (EGF), leptin, and insulin (17). RTKs are single-span transmembrane receptors that facilitate communication between cells and their extracellular environment. Tyrosine phosphorylation (pTyr), a crucial post-translational modification, regulates essential biological processes that include cellular proliferation, migration, and invasion (24). The process is mediated by the coordinated actions of both protein tyrosine kinases (PTKs), which add phosphate (PO_4_) groups, and PTPs, which remove these phosphate groups (25, 26). Disruption of this delicate balance between PTKs and PTPs can result in aberrant pTyr. While most previous research historically focused on the role of PTKs, recent studies have emphasized the critical functions of PTPs in maintaining this balance. As PTPs can act as both initiators and terminators of pTyr signaling, their importance in maintaining cellular homeostasis is paramount (27, 28); absence or dysfunction of PTPs can lead to a myriad of pathological conditions, including autoimmunity, cancer, CVD, and obesity-related metabolic disorders (29, 30).

### Insulin signaling and the role of PTP1B

In healthy individuals, food intake elevates blood glucose and stimulates insulin release from the pancreas, inducing a feedback mechanism that increases glucose uptake by peripheral tissues to regain normal blood glucose concentrations (31, 32). Insulin resistance occurs when tissues such as skeletal muscle, adipose tissue, and the liver become less responsive to insulin, leading to reduced glucose uptake, hyperglycemia, and hyperinsulinemia, and eventually, T2D (33, 34).

**Figure 1:**
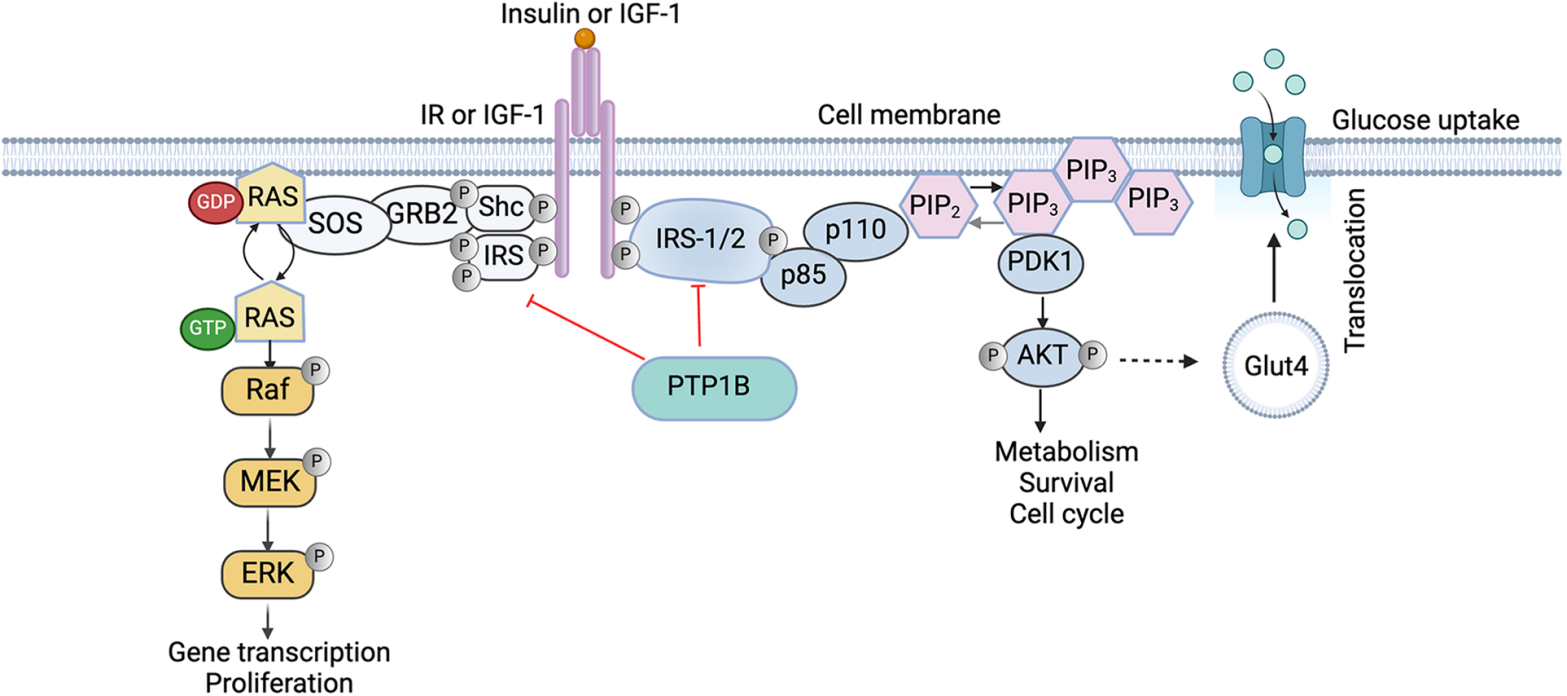
Role of PTP1B in the insulin signaling cascade. Binding of Insulin receptor substrates (IRS) activates the phosphatidylinositol-4,5-biphosphate-3-kinase (PI3K)/AKT pathway which is crucial for cellular survival and the mitogen-activated protein kinase (MAPK) pathway that is responsible for cell differentiation and cell growth. PTP1B negatively regulates insulin signaling by directly dephosphorylating both the IR and IRS1/2, reducing the activity of downstream effectors, including the PI3K/AKT pathway, reducing cellular glucose uptake. IR, insulin receptor; IGF, insulin-like growth factor; PTP1B, protein tyrosine phosphatase 1B; MEK, MAPK ERK kinase; ERK, extracellular signal-regulated kinases; p85 and p110, subunits of PI3K; PIP, phosphatidylinositol phosphate; PDK, protein dependent kinase. Image created with BioRender.com.

Insulin signaling, a key RTK pathway, and PTP1B regulation are integral mediators of this biological process. Briefly, when insulin is released into the bloodstream by the pancreas, it binds to cells through the insulin receptor (IR), inducing autophosphorylation and activation of its intrinsic kinase activity (35). The activated receptor then phosphorylates its downstream effectors, the insulin receptor substrates (IRS1/2), which mediate activation of phosphatidylinositol (PI) 3′-kinase (PI3K), a lipid kinase complex consisting of a regulatory p85 subunit and a catalytic p110 subunit that phosphorylates the 3′-hydroxyl group of phosphoinositides. Specifically, with respect to insulin signaling, phosphorylated IRS proteins interact with the regulatory p85 subunit of PI3K through their SH2 domains, inducing a conformational change that relieves the inhibition on the p110 catalytic subunit, recruiting the now activated PI3K complex from the cytosol to the plasma membrane (PM). Once at the membrane, PI3K can phosphorylate its substrate, phosphatidylinositol 4,5-bisphosphate [PtdIns(4,5)P2, also known as PIP2] to generate phosphatidylinositol-3,4,5-trisphosphate (PIP3), driving downstream signaling and activation of protein kinase B (PKB; also known as AKT) (36). AKT then further promotes activation of downstream effectors involved in protein synthesis and survival, as well as induces translocation of glucose transporters to the cell membrane, resulting in increased glucose uptake by the cells (37) (Figure 1).

PTP1B negatively regulates this process by directly dephosphorylating both the IR and its downstream effectors, IRS1 and IRS2, reducing their activity and downregulating PI3K/AKT signaling (38). Indeed, whole-body PTP1B knockout (KO) mice exhibit enhanced insulin sensitivity, elevated phosphorylation of IR and IRS-1, and resistance to high-fat diet (HFD)-induced weight gain and obesity (27). Functionally, PTP1B KO mice have elevated basal metabolic rates and increased energy expenditures (39). Interestingly, the effects of reduced adiposity are not consequent to decreased fat cell numbers; rather, they are mediated by having decreased fat cell sizes (39). Collectively, these findings demonstrate a critical role for PTP1B in the regulation of metabolism *in vivo*.

### PTP1B regulation of leptin signaling

**Figure 2:**
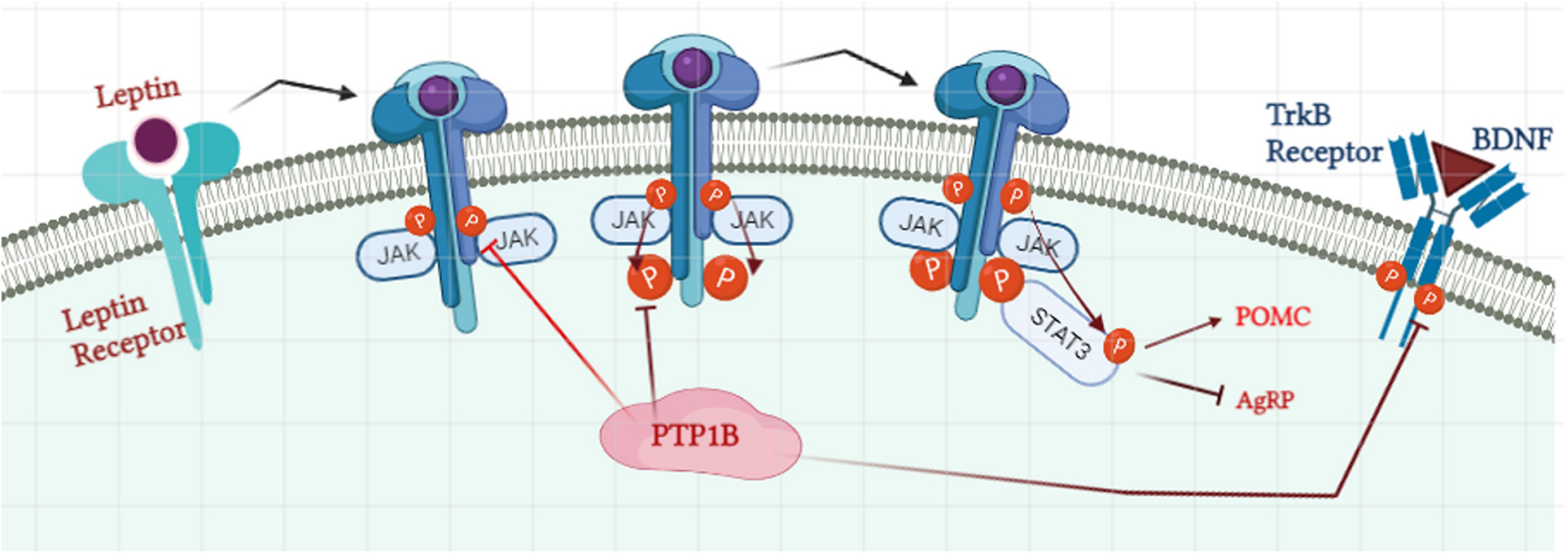
Role of PTP1B in regulation of the leptin signaling pathway. Binding of leptin to the leptin receptor initiates the recruitment of Janus Kinase 2 (JAK2) and their auto-phosphorylation process. Phosphorylated JAK2 then phosphorylates the tyrosine residue on the receptor which allows the docking of signal transducer and activator of transcription 3 (STAT3). STAT3 promotes the activation of Proopiomelanocortin (POMC) and inhibits the expression of agouti-related protein (AgRP). PTP1B suppresses the phosphorylation level of JAK2 and leptin receptors and henceforth STAT3 docking. PTP1B: protein tyrosine phosphatase 1B. Image created with BioRender.com.

Leptin, an adipocyte-secreted hormone, acts in the mediobasal hypothalamic nuclei to suppress food intake and increase energy expenditure, regulating energy balance and body mass (40). As such, circulating leptin levels correlate with adiposity (41). Mechanistically, leptin binds to the leptin (LR), a type I cytokine receptor that is expressed in the hypothalamus, and activates its effector JAK2, a protein that promotes the growth and division of cells (42). Once activated, JAK2 induces activation of the IRS proteins, which like insulin signaling, can lead to the activation of the PI3K/AKT pathway (43) (Figure 2). Activation of JAK2 also directly phosphorylates the LR itself, allowing for recruitment of the SH2 domain-containing protein-tyrosine phosphatase 2 (SHP2), another critical PTP that promotes activation of the extracellular signal-regulated kinase 1/2 (ERK1/2)-mitogen activated protein kinases (MAPK) pathway, which is critical for the proliferation and growth of cells (44, 45). In parallel, JAK2-mediated LR phosphorylation also drives activation of the signal transducer and activator of transcription factor 3 (STAT3) pathway, which suppresses transcription of both the hypothalamic neuropeptide Y (Npy) and agouti-related protein (Agrp), co-expressive neuropeptides that stimulate food intake and repress energy expenditure (46).

Like with insulin, PTP1B is a negative regulator of leptin signaling by directly dephosphorylating JAK2, thereby blocking the phosphorylation and activities of both the receptor and STAT3 (47). PTP1B KO mice have increased leptin sensitivity, lower leptin/body fat ratios, and enhanced hypothalamic signaling, leading to reduced adiposity and increased energy expenditure (48).

## Effects of PTP1B in metabolism

Located on chromosome 20q13.1-13.2, several single nucleotide polymorphisms (SNPs) in the *PTPN1* gene have been linked to obesity and diabetes in various populations. In French Canadians, for example, at least six PTP1B SNPs have been associated with these conditions (49, 50). PTP1B SNPs have also been identified in individuals with early onset diabetes in Danes, Canadians, and Italians (10, 51, 52). Moreover, the PTP1B SNP rs3787348 is linked to a poorer weight reduction outcome in obese Japanese patients (53). Based on this, and the PTP1B global KO mouse data, it is clear that PTP1B influences glucose homeostasis and energy balance through its actions on insulin and leptin pathways. Inhibition of PTP1B enhances signaling to these pathways, improving glucose uptake and energy expenditure, and thus highlights the crucial role for PTP1B in metabolic health.

However, despite these overall positive outcomes, global, as well as myeloid-specific, PTP1B KO mice can develop acute myeloid leukemia and have been shown to have reduced lifespans (27, 54), suggesting differential tissue-specific effects of PTP1B. As such, global therapeutic targeting of PTP1B, particularly for treatment of metabolic diseases, appears to be rather complicated. To therefore circumvent this issue and to better elucidate the precise molecular, functional, and biological regulations of PTP1B, tissue-specific deletion models for PTP1B have been generated and studied (Table 1).

**Table 1:** Effects of global and tissue specific deletion of PTP1B in rodents.

Site of deletion	Genotype	Observed phenotype	References
Global	PTP1B^−/−^	1. Lower blood glucose concentrations 2. Enhanced insulin sensitivity 3. Increased basal metabolic rate and total energy expenditure 4. Resistant to weight gain and remained insulin sensitive on HFD	(27, 55)
Muscle	PTP1B^−/−^	1. Increased muscle glucose uptake 2. Improved systemic insulin sensitivity 3. Enhanced glucose tolerance	(56, 57)
Adipocyte	PTP1B^−/−^	1. No differences in body weight/adiposity compared to PTP1B^+/+^ 2. Increased adipocyte size 3. Increased circulating glucose and leptin levels 4. Reduced leptin sensitivity 5. Increased basal lipogenesis	(58, 59)
Neuronal	PTP1B^−/−^	1. Reduced weight and adiposity 2. Increased energy expenditure 3. Hypersensitive to leptin and elevated leptin levels 4. Improved glucose homeostasis	(57, 60)
POMC	PTP1B^−/−^	1. Reduced adiposity 2. Improved leptin sensitivity 3. Increased energy expenditure 4. Reduced BP response to ganglionic blockade 5. Increased vascular adrenergic reactivity 6. Exhibited a blunted increased in diastolic and mean BP	(61)
Myeloid	PTP1B^−/−^	1. Impaired BMDC activation 2. Decreased migratory capacity of epidermal DC 3. Fail to present antigen to T cells as efficiently as control BMDC 4. Shortened lifespan and development of acute leukemia	(54)
Liver	PTP1B^−/−^	1. Improved glucose homeostasis and lipid profiles 2. Increased hepatic insulin signaling 3. Decreased triglyceride and cholesterol levels 4. Protective effect against endoplasmic reticulum stress	(62, 63)
Endothelial cell	PTP1B^−/−^	1. Promotes cardiac and extracardiac angiogenesis during chronic pressure overload-induced hypertrophy 2. Reduce hypoxia, oxidative stress and fibrosis 3. Enhanced cardiac VEGF signaling and increased Caveolin-1 expression 4. Restore altered NO production in the peripheral circulation in CHF	(64–66)
Islet	PTP1B^−/−^	1. Absence of islet PTP1B increased graft functional vascularization by increasing the number of newly formed vessels 2. Up-regulation of several EC markers and reduced IEC loss in culture 3. Enhanced VEGF-A production and activation of the PGC1α/ERRα axis	(67)

HFD, high fat diet; BP, blood pressure; POMC, pro-opiomelanocortin; BMDC, bone marrow dendric cells; DC, dendric cell; VEGF, vascular endothelial growth factor; NO, nitric oxide; CHF, chronic heart failure; EC, endothelial cell; IEC, induced endothelial cell.

### Skeletal muscle

PTP1B deletion in skeletal muscle cells enhances systemic insulin sensitivity and increases glucose uptake in the muscle, independent of body weight and adiposity (56). These mice also exhibit increased insulin signaling, as indicated by enhanced phosphorylation of the IR and its downstream effector IRS1, as compared to controls (56). PI3K activity and phosphorylation of both AKT and ERK1/2 are also elevated in these mice. Despite these effects, however, deficiency of PTP1B in skeletal muscle does not affect lipid profiles, leptin levels, leptin sensitivity, body weight, or adiposity (56, 68), suggesting that these mechanisms for PTP1B regulation reside in other tissues and/or cell types.

### Adipose

Adipose-specific PTP1B KO mice maintain similar body weight to controls when fed a HFD, but have larger adipocytes, increased circulating leptin levels, and increased basal lipogenesis (58). These mice have decreased phosphorylation of IR and its downstream effector AKT and have overall increased expression of lipogenic genes (58). Moreover, pyruvate kinase M2 (PKM2) is a newly identified substrate for PTP1B in adipocytes, but its mechanism of regulation in these cells remains unclear (69). In addition, PTP1B's role in different adipose depots is variable, influencing adipogenesis and insulin sensitivity differently between white and brown adipocytes; specifically, PTP1B knockdown in 3T3-L1 adipocytes inhibits adipogenesis (69), whereas PTP1B deficiency in brown adipocytes accelerates adipogenesis and enhances insulin sensitivity through activation of AKT and increased resistance to TNF*α*-induced apoptosis (70, 71).

### Liver

Mice with liver-specific PTP1B deletion show increased insulin sensitivity and suppressed hepatic gluconeogenesis and lipogenic genes, without affecting weight gain (62, 63). In addition, these mice have alleviated endoplasmic reticulum (ER) stress following HFD, with decreased phosphorylation of downstream p38-MAPK and c-Jun N-terminal kinase (JNK)-MAPK signaling pathways, as well as reduced ER-resident proteins, PERK and eukaryotic initiation factor 2 α (eIF2α) factors (62, 63). This decreased signaling also leads to lowered expression of stress-related transcription factors and improved lipid profiles (62, 63). Liver-specific PTP1B KO, however, does not impact leptin levels, leptin sensitivity, body weight, or adiposity (62, 63), underscoring the liver-specific benefits of PTP1B inhibition in metabolic regulation.

### Neuronal

Neuronal deletion of PTP1B in mice show decreased body mass and adiposity, as well as improved glucose homeostasis (57). These mice also exhibit increased physical activity and energy expenditure and are hypersensitive to leptin with elevated leptin levels (57). Moreover, deletion of PTP1B in arcuate pro-opiomelanocortin (POMC), a specific regulatory neuronal subpopulation that controls metabolism and is expressed only in hypothalamus and regions of the spinal cord and brain stem, reduces adiposity, improves leptin sensitivity, and increases energy expenditure (61, 72). These mice are also protected against leptin- and sympathetically mediated increases in blood pressure. Collectively, these data suggest that PTP1B modulates leptin production and resistance primarily through activation of pathways within the central nervous system (CNS) (61). In addition, POMCs induce brain-derived neurotrophic factor (BDNF) signaling in the hypothalamus, mediating neural connectivity and neurite outgrowth (73). In this regard, PTP1B modulates this process by acting as a direct negative regulator of the downstream BDNF effector, tropomyosin receptor kinase B (TrkB) (60, 73, 74), thus increasing both AKT and ERK activation. These effects highlight the critical role of the CNS in PTP1B-mediated metabolic regulation and the control of food intake and energy expenditure.

## PTP1B in CVDs

As previously discussed, obesity is a major risk factor for CVD, increasing mortality and morbidity, particularly in individuals with central adiposity (75). Obesity is also associated with the development of high blood pressure, a leading risk factor for stroke, cardiac hypertrophy, and heart failure (76, 77). Several studies have established a significant link between PTPs, PTKs and cardiac pathophysiology (78). Indeed, increased activation of PTP1B in the heart is associated with development of heart failure in both rats and humans (78, 79). Cardiac contractile dysfunction and intracellular calcium dysregulation are also linked to elevated levels of PTP1B (80). Thus, there is precedence for a critical role for PTP1B in CVD (81).

### PTP1B and endothelial cells (ECs)

Endothelial dysfunction is a primary event in the progression of CVD and is a known predictor of diabetes- and obesity-related CVD (82). Increased PTP1B expression in ECs impairs macro- and micro-vascular systems and increases the risk of having an MI or ischemic stroke (64, 83–87). Mechanistically, enhanced PTP1B-mediated signaling in ECs inhibits activation of AKT and blocks endothelial nitric oxide synthase (eNOS), an enzyme responsible for producing nitric oxide (NO), a potent vasodilator and regulator of glucose uptake (64, 88). Inhibition of PTP1B enhances serine phosphorylation of eNOS and improves NO-mediated vasodilation in mice with chronic heart failure induced by coronary ligation (64). These data suggest that PTP1B inhibition could potentially be considered for patients with CVD and/or endothelial dysfunction.

**Figure 3:**
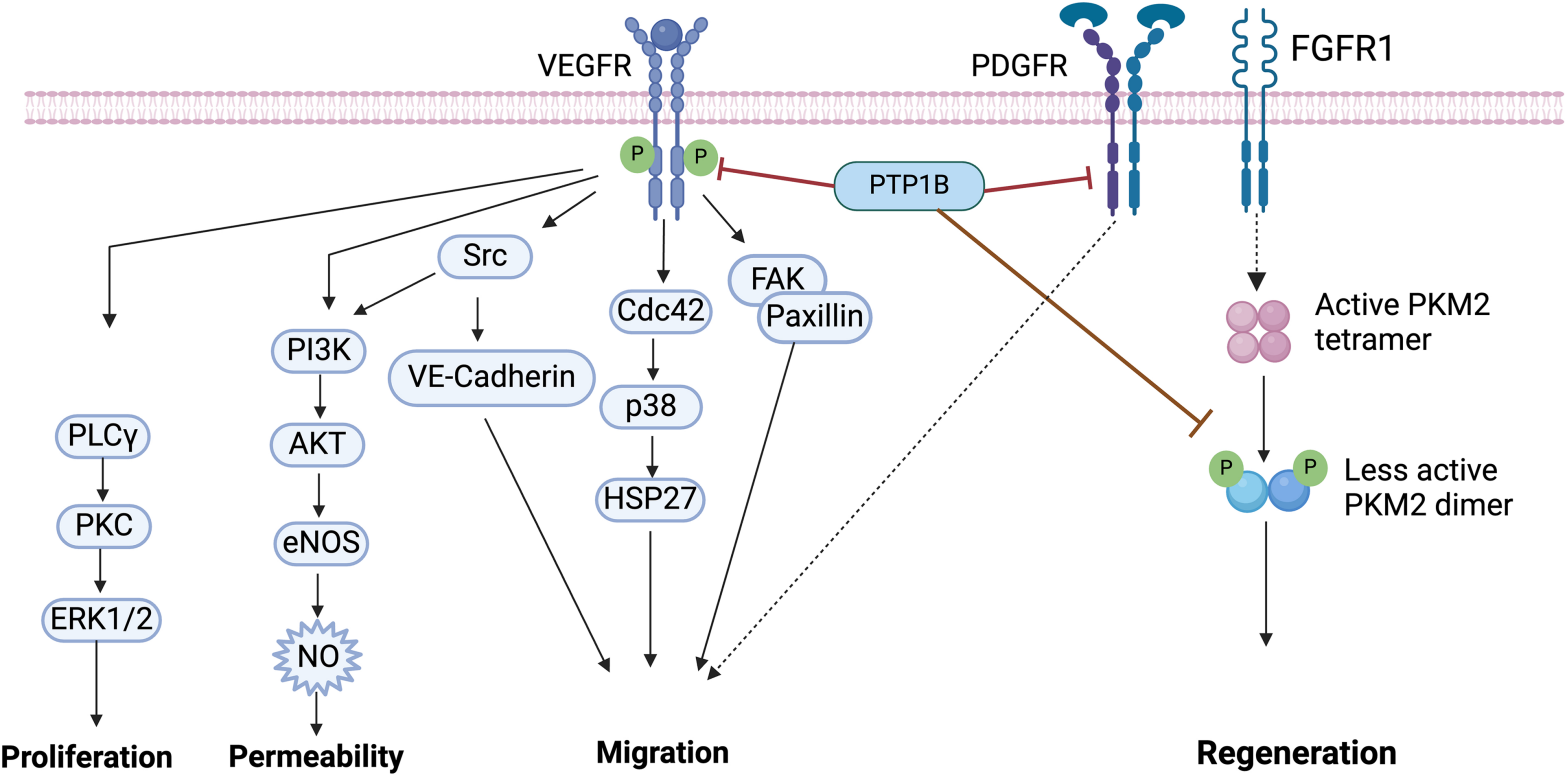
Vascular endothelial growth factor (VEGF) signaling. Binding of VEGF to VEGFR induces its tyrosine phosphorylation, which is followed by the activation of downstream effectors including PLCγ/PKC/ERK1/2, PI3K/AKT, Src, p38 and FAK/Paxillin. PTP1B binds to VEGFR and directly dephosphorylates VEGFR. PTP1B directly binds to platelet-derived growth factor receptor (PDGFR), inducing motility in smooth muscle cells (SMCs). The fibroblast growth factor receptor (FGFR1) directly phosphorylates pyruvate kinase M2 (PKM2), PTP1B dephosphorylates PKM2 and regulates myocyte regeneration. VEGF, vascular endothelial growth factor; PTP1B, protein tyrosine phosphatase 1B; FAK, focal adhesion kinase; PI3K, phosphatidylinositol 3-kinases; eNOS, endothelial nitric oxide synthase; NO, nitric oxide; PLCy, phospho-lipase C gamma; PKC, protein kinase C; ERK1/2, extracellular signal-regulated kinase 1/2; PKM2, pyruvate kinase M2. Image created with BioRender.com.

Vascular endothelial growth factor (VEGF) receptor 2 (VEGFR2) and its ligand VEGF-A are key mediators of angiogenesis, the process by which existing blood vessels grow (89). Binding of VEGF-A to VEGFR2 induces autophosphorylation of the receptor and, subsequently, leads to activation of multiple crucial downstream signaling pathways necessary for survival, proliferation, and migration of ECs (90). To drive angiogenesis, VEGFR signaling activates Src, which induces phosphorylation of VE-Cadherin, as well as P38-MAPK, to mediate EC migration (91). In addition, VEGFR activation induces signaling to both ERK1/2 and PI3K/AKT, to promote EC proliferation and EC survival, respectively (92) (Figure 3).

Here too, PTP1B has a prominent role in CVD; it directly dephosphorylates VEGFR2 to inhibit angiogenesis (66). Mice with endothelial-specific deletion of PTP1B, however, have improved angiogenesis, enhanced wound healing, and are protected against development of chronic heart failure post-MI (66, 93). Primary ECs isolated from these mice reveal that the mechanism of this regulation is mediated by enhanced VEGF-A-induced VEGFR2 activity, which leads to increased ERK signaling to induce angiogenesis (66). Similar effects on angiogenesis, revascularization and survival are also noted in PTP1B-deleted pancreatic islets (67), as well as in human umbilical vein ECs, both *in vitro* and *in vivo* (94).

### PTP1B and vascular smooth muscle cells (VSMCs)

The motility and proliferation of VSMCs is critical for neointima formation and remodeling in response to vascular injury (95). Elevated levels of platelet-derived growth factor (PDGF) and/or fibroblast growth factor-2 (FGF2) contribute to this remodeling event, mediating autophosphorylation and activation of PDGF receptor β (PDGF-βR) and increased downstream signaling (96). Here, induction of NO can increase PTP1B activity, whose direct binding to the PDGF-βR can then inhibit downstream signaling and suppress proliferation and motility in cultured SMCs (96–98).

Indeed, adenoviral overexpression of PTP1B blocks insulin-mediated activation of IR and decreases cell motility, whereas dominant-negative expression of PTP1B attenuates NO-mediated inhibition of cell motility (98). Treatment of injured arteries with dominant negative PTP1B adenovirus, however, increases cell proliferation, intimal cell number, and neointima formation, without affecting apoptosis. Moreover, primary isolated newborn rat aortic SMCs treated with an antisense oligonucleotide against PTP1B enhances cell motility and increases pTyr of several adhesion molecules, including p130^cas^, paxillin and focal adhesion kinase (FAK) (99) (Figure 3). Together, these findings demonstrate that PTP1B is an important regulatory protein involved in motility and proliferation, and that it may be a good potential therapeutic target for treating VSMC pathophysiology.

### PTP1B and cardiac myocytes

Cardiomyocytes (CMs) are contractile muscle cells that allow the heart to maintain proper pumping function (100). CMs are also the major source of VEGF signaling in the heart (101, 102). Here, activation of P38-MAPK promotes secretion of VEGF-A, which in turns acts as a feedback mechanism to stimulate binding and activation of the VEGFR (103). CM-mediated VEGF signaling also mediates cardiac morphogenesis, transforming cardiac ECs into mesenchymal cells in mice (104, 105). However, the mechanisms for how PTP1B is involved in this process in CMs requires further study.

Interestingly, research on PTP1B activity in CMs has demonstrated both protective and deleterious effects. For example, PTP1B directly binds PKM2, increasing its activity in CMs (106). Since PKM2 is a key cell cycle regulator, increasing its activity induces CM cell division, improves cardiac function, and extends long-term survival after acute or chronic MI (107) (Figure 3). Conversely, PTP1B overexpression mediates age-induced CM contractile dysfunction and other myocardial anomalies (80, 108).

In response to HFD, global deletion of PTP1B in mice reduces cardiac pathologies, including hypertrophy (108). Our own laboratory also recently demonstrated that CM-specific deletion of PTP1B in mice ameliorates HFD-induced cardiomyopathy and cardiac steatosis (106). Furthermore, metabolomics data revealed that CM-specific deletion of PTP1B elevates fatty acid oxidation and lipolysis, but reduces glucose metabolism (106). In response to hypoxia-reoxygenation of isolated primary neonatal mouse CMs, PTP1B expression increases; ablation of PTP1B in these cells increases AKT activity and decreases hypoxia-associated apoptosis (109). These findings indicate that PTP1B inhibition may mitigate cardiac abnormalities and improve heart function.

While these data all suggest a protective role for PTP1B deletion in the heart, Coulis et al. have found that CM-specific deletion of PTP1B in mice may also be pathological, inducing a hypertrophic phenotype that is exacerbated by pressure overload (110). Specifically, they found that argonaute 2 (AGO2), a critical component of the RNA-induced silencing complex, is inactivated in response to CM-specific deletion of PTP1B, thereby preventing miR-208b-mediated inhibition of mediator complex subunit 13 (MED13) and leads to thyroid hormone-associated pathological cardiac hypertrophy (110). However, in this regard, inhibition of miR-208b has previously been shown to improve, not induce, cardiac dysfunction in titin-induced dilated cardiomyopathy (111). Moreover, upregulation of MED13 is protective in the heart, conferring resistance to obesity (112). Regardless, it remains possible that different pathological stimuli may lead to different outcomes and/or that too much or too little PTP1B can lead to similar, but distinct, pathological outcomes (106). Exactly if and how PTP1B is involved in modulating these various physiological vs. pathological responses in the heart remains to be determined (106).

## PTP1B as a potential therapeutic target

Overall, given PTP1B's critical role in insulin and leptin signaling, it is considered an excellent target for treating obesity and diabetes (17). However, targeting PTP1B, in a cellular and tissue-specific manner, has been challenging, particularly because of its positively charged active site, its selectivity, and its bioavailability (78, 113). Consequently, only a few PTP1B inhibitors have been tested in clinical trials, and most have, unfortunately, been discontinued due to insufficient efficiency, lack of specificity and detrimental side effects (114–116) (Table 2). Therefore, more research is needed to fully elucidate the effects of PTP1B inhibition in cardiometabolic diseases.

**Table 2:** An overview of PTP1B inhibitors.

PTP1B inhibitor	Inhibitor type	In diabetic & cardiovascular studies	IC50 value	References
MSI-1436/trodusquemine	Noncompetitive, reversible, allosteric inhibitor	1. Reduced fat & insulin levels in HFD induced obese mice 2. Weight loss, suppressed food intake, decreased adipocyte size/lipid content & lowered plasma insulin levels 3. Reduced osteogenic & myofibrogenic VIC differentiation 4. Decreased VIC osteogenic differentiation, inhibits myofibroblast, & protective against aortic valve stenosis 5. Decreased phosphorylation of p44/42 MAP kinase in breast cancer model mice	0.6 μM–40.6 μM, depending on PTP1B enzyme form	(117, 118)
ISIS 113715	Antisense inhibitor	1. Inhibited PTP1B mRNA and protein expression in monkey hepatocytes 2. Reduced PTP1B mRNA expression in liver/adipose tissues in monkeys 3. Increased adiponectin concentrations & lowered insulin responses in monkeys 4. Improved insulin sensitivity & normalized plasma glucose levels in mice and monkeys 5. Downregulated lipogenic genes within fat and liver as well as adipocyte differentiation associated genes in fat tissue of mice 6. Tolerated and safe in people, improved dyslipidemia & increased adiponectin, decreased body weight	<0.01 μM	(59, 119–121)
BDB	Competitive, selective inhibitor	1. Antidiabetic effects in spontaneously developing diabetic mice. 2. Marked improvements in pancreatic islet cell architecture and increased ratios of β-cells to α-cells	1.86 μM	(87)
DPM-1001	Analog of MSI-1436	1. Reversed obesity 2. Improved glucose tolerance 3. Insulin sensitivity	0.1 μM	(122)
Isoxazol-5 (4H) one derivative C3	Competitive, binds to the active site	1. Suppressed weight gain in mice upon HFD	2.3 µM	(123)
TCS 401	Competitive, selective inhibitor	1. Increased dopamine response to insulin & general responsiveness to insulin compared to the HFD control mice 2. Increased ERK & AKT expression in RPE & CMECs 3. Decreased apoptosis & caspase 3 activity in CMEC	2 μM	(124–127)
XWJ24	Competitive and selective inhibitor, 4.5 times more potent than TC-PTP	No studies in the context of obesity, diabetes or cardiovascular disease	0.6 μM	(128)

HFD, high fat diet; VIC, valvular interstitial cells; RPE, retinal pigment epithelial; CMEC, cardiac microvascular endothelial cell.

Ertiprotafib, a monocarboxylic acid mimetic, is a noncompetitive multiple-action inhibitor (129). It was the first PTP1B inhibitor to be tested in clinical trials for the treatment of diabetes (130). This inhibitor has an atypical mechanism of action; while most small molecules bind to and increase their target's melting temperature (Tm) to stabilize their interaction, ertiprotafib lowers the Tm of PTP1B, destabilizing it (131, 132). Indeed, the mechanism by which ertiprotafib inhibits PTP1B activity is mediated by inducing protein aggregation (133). However, the selectivity of this compound is very low, with IC50 values of 1.6–29 µM, depending on assay conditions (129). Therefore, development of newer and more specific inhibitors was needed.

Trodusquemine, also known as MSI-1436, is a natural aminosterol cholestane that is a noncompetitive, reversible, and allosteric inhibitor of PTP1B (117, 134). In terms of specificity, this small molecule targets a novel allosteric binding site located within the C-terminus of PTP1B, allowing it to bind with affinity values that range from 0.6 μM to 40 6 μM, depending on the enzyme form (117). Because of its high specificity, trodusquemine was initially considered to be the more effective inhibitor with fewer off-target side effects (135). Indeed, in HFD-fed mice, MSI-1436 significantly decreased obesity by reducing insulin levels, decreasing body weight, lowering lipid content, decreasing adipocyte size, and suppressing food intake (118). In addition, MSI-1436 reduced osteogenic and myofibrogenic valvular interstitial cell (VIC) differentiation, alleviating aortic valve fibro-calcification and stenosis in a diet-induced mouse model of calcific aortic valve disease (136). Unfortunately, though effective in these mouse studies, this inhibitor was discontinued for use in human clinical trials due to its low activity and poor bioavailability in patients (19).

JTT-551, a mixed-type PTP1B inhibitor, was shown to have good selectivity. Like trodusquemine, chronic administration of JTT-551 in mice with diet-induced obesity demonstrated anti-obesity properties (137). In addition, JTT-551 reduced blood glucose and exhibited antidiabetic effects, without changes in body weight, in diabetic mice (137). Taken together, these data indicated that JTT-551 was an effective inhibitor for treatment of T2D and obesity; however, trials here too were discontinued due to insufficient efficacy and increased adverse effects in patients (138).

### New therapies on the horizon for PTP1B inhibition

Fortunately, several new PTP1B inhibitors have been recently generated and are in process of being tested for efficacy against a myriad of cardiometabolic diseases, including T2D. The hope is that one (or several) will succeed in patient trials and provide future therapeutic benefits for these devastating diseases.

IONIS-PTP-1BRx, also known as ISIS-113715, is an antisense PTP1B inhibitor with an IC50 of less than 0.01 μM (59). In obese insulin-resistant monkeys, this inhibitor improved insulin sensitivity, increased adiponectin concentration, and lowered insulin responses (59). Similarly, in insulin-resistant diabetic mice, IONIS-PTP-1BRx improved insulin sensitivity, normalized plasma glucose levels, and decreased expression of lipogenic genes in both fat and liver, downregulating adipocyte differentiation (120). In 2018, IONIS-PTP-1BRx made its way to clinical trials, and preliminary data indicate that treated T2D diabetic patients have reduced body weight and decreased mean HbA1c levels (121), all without adverse interactions with other antidiabetic drugs (119). However, as of the date of this submission, no further updates have been posted about this study.

BDB [3-bromo-4,5-bis(2,3-dibromo-4,5-dihydroxybenzyl)-1,2-benzenediol] is a recently developed competitive inhibitor of PTP1B with demonstrated high selectivity (87). *In vitro*, BDB is cell-permeable and enhances insulin signaling. Moreover, oral administration of BDB leads to antidiabetic effects in spontaneously developing diabetic mice, with marked improvements in pancreatic islet cell architecture and increases in ratios of β-cells to α-cells (87). No information, however, is yet available about its progression to clinical trials.

DPM-1001, an analog of MSI-1436 with an IC50 of 0.1 μM, reverses obesity and improves insulin sensitivity and glucose tolerance in HFD mice (139). In May of 2022, DepYmed released a statement that it received FDA orphan drug designation for the clinical candidate DPM-1001, for the treatment of Wilson Disease, a rare genetic disorder that prevents the body from removing copper, causing the metal to build up in the liver, brain, and corneas (122) (https://www.iospace.com/article/releases/depymed-receives-fda-orphan-drug-designation-for-clinical-candidate-dpm-1001-for-the-treatment-of-wilson-disease/). This classification for DPM-1001 is an important milestone, as it furthers the development and progression of PTP1B inhibitors for use in diseased patients.

Other potent inhibitors have also been recently studied in the context of metabolism. A competitive derivative of Isoxazol-5(4H) suppresses weight gain in HFD mice, although at a comparatively reduced potency than other PTP1B inhibitors (123). TCS 401, another competitive and selective inhibitor, increases dopamine responses to insulin in HFD mice (124). Inhibitors, such as XWJ24 and compounds from D. crassirhizomai and M. alba, have recently shown strong PTP1B inhibition, but have yet to be tested in HFD or CVD studies (128, 140, 141). Finally, a new patent application has identified 5-(naphthalen-2-yl)-1,2,5-thiadiazolidin-3-one 1,1-dioxide derivatives (represented by “formula 1”) as a newly garnered PTP1B inhibitor, but its efficacy and specificity have yet to be determined (114).

## Navigating the challenges: future opportunities for PTP1B inhibitors in disease treatment

### New targeting strategies for PTP1B inhibition

The transcriptional and translational regulation of PTP1B is well studied, providing potential novel sites for therapeutic targeting, particularly for metabolic disorders and cancers. In this regard, characterization of the PTP1B promoter region revealed several binding sites for transcription factors such as Egr-1, Sp1, Sp3 (142), YB-1 (143), NF-κB (144) and HIF (145). These factors can be activated by various stimuli, including glucose, insulin, proinflammatory cytokines, and oncoproteins (146). Additionally, PTP1B expression is regulated by several miRNAs, including miR-338-3p, miR-744, miR-122, miR-193a-3p, miR-135a, miR-146-b, and miR-206 (147–152). Each of these can be considered as additional, indirect PTP1B inhibitors for future studies, as they have the potential to be targeted with high specificity, to reduce downstream PTP1B signaling, and hopefully, to prevent development of insulin resistance, T2D, CVD, as well as onset of other diseases such as cancer. The efficacy of this strategy remains to be determined.

### Outside the box approaches for PTP1B inhibition

Over the past few decades, numerous PTP1B inhibitors have been identified, but their lack of specificity and cellular permeability pose significant challenges for clinical use (115). Most inhibitors target the catalytic active site, leading to non-specific inhibition of all PTPs (153). Despite efforts to develop more specific inhibitors, the high conservation of the catalytic site among PTPs, particularly TCPTP, remains a major obstacle. To overcome these challenges, researchers are exploring innovative strategies including high-throughput screening (154), virtual screening (155), DNA-encoded libraries (156), and PROTAC approaches (157) to develop drugs with greater efficacy and increased specificity. Additionally, designing inhibitors with improved membrane permeability and bioavailability is crucial. In this regard, nanoparticle-based delivery systems and graphene quantum dots show promise in enhancing oral bioavailability and specificity (158, 159). Addressing these challenges will be necessary for developing effective and safe PTP1B inhibitors for clinical treatment of various diseases in the near future.

## Summary

PTP1B signaling is a critical regulator of glucose homeostasis and energy balance, conducive to mediating the onset of obesity, insulin resistance, T2D, and CVD (49, 160). Consequently, inhibition of PTP1B is an attractive novel strategy for treating these diseases. Though significant progress has been made in understanding the molecular mechanisms of PTP1B regulation in metabolic processes that affect diabetes, obesity and the heart, tissue-specific targeting and selective potency with existing PTP1B inhibitors remains challenging. Future research is promising in this regard, and advancements in this field, through novel and targeted modulation of PTP1B, are likely to yield effective results and therapies for treatment of individuals with these devastating ailments.
